# Macrodystrophia Lipomatosa: A Rare Case of Right Lower Extremity Gigantism Associated With Syndactyly

**DOI:** 10.1002/ccr3.72036

**Published:** 2026-02-24

**Authors:** Hafiza Sameeya Shehzadi, Saumara Atif, Iqbal Hussain Dogar, Usama Siddique, Kamil Ahmad Kamil

**Affiliations:** ^1^ Senior Registrar Radiology Department Gujranwala Medical College Teaching Hospital Gujranwala Pakistan; ^2^ Radiology Department Gujranwala Medical College Teaching Hospital Gujranwala Pakistan; ^3^ Head of Department of Radiology Gujranwala Medical College Teaching Hospital Gujranwala Pakistan; ^4^ Faisalabad Medical University Faisalabad Pakistan; ^5^ Mirwais Regional Hospital Kandahar Afghanistan

**Keywords:** entire right lower extremity, Macrodystropic lipomatosa, MRI, syndactyly

## Abstract

Macrodystrophia lipomatosa (MDL) is a rare sporadic, nonhereditary developmental condition, typically presenting at birth or in early childhood. It is characterized by progressive overgrowth of tissues, mainly due to excess fibrofatty tissue proliferation. This abnormal growth commonly involves nerve sheath, muscle, periosteum, and bone marrow. Herein, we present a case of a 10‐month‐old boy who was referred to our institution for evaluation of unilateral right lower limb enlargement. Clinical examination revealed soft tissue hypertrophy and syndactyly of the right lower limb. To assess the extent of limb involvement and differentiate MDL from other causes of overgrowth syndromes, a series of imaging studies was conducted. Upon confirmation of diagnosis, the patient was referred to the pediatric surgery department. The study highlights the characteristic clinical and imaging features of MDL, with particular emphasis on the MRI findings, which are essential for accurate diagnosis and differentiation from other causes of limb overgrowth.

## Key Clinical Message

Macrodystrophia Lipomatosa (MDL) is a rare condition that may present with progressive limb overgrowth and syndactyly. Early imaging with MRI is essential for distinguishing MDL from other causes of overgrowth syndromes and guiding appropriate management.

## 1 Introduction

Macrodystrophia lipomatosa (MDL) is a rare, nonhereditary disorder that typically presents at birth or during the neonatal period, characterized by progressive overgrowth of mesenchymal tissues, particularly a disproportionate increase in fibrofatty components. It was first reported by Feriz in 1926 [1]. It is associated with other anomalies including polydactyly, brachydactyly, syndactyly, and symphalangism. Mostly, overgrowth is unilateral and localized in the fingers or toes. Distal limb involvement is typically observed in MDL, with the lower limbs being more commonly affected than the upper limbs [2]. There is no significant gender predilection; however, a slight male predominance has been reported in the literature [3]. MDL is considered to fall under the umbrella of a broader spectrum of PIK3‐related overgrowth syndromes (PROS), associated with mutations in the PIK3CA gene leading to abnormal signaling and overgrowth of mesodermal and ectodermal tissues. Fibrofatty tissue proliferation is one of the manifestations seen in PROS and is the hallmark of MDL. Other conditions associated with overgrowth and related to PIK3CA gene mutation can manifest in syndromic forms or as isolated manifestations. PROS syndromes include CLOVES syndrome, KTS syndrome, macrocephaly‐capillary malformation, fibroadipose overgrowth, hemihyperplasia‐multiple lipomatosis, and facial infiltrating lipomatosis. Whereas isolated manifestations can occur in forms of macrodactyly, hemimegalencephaly, dysplastic megalencephaly, focal cortical dysplasia (FCD) type IIa, muscular hemihyperplasia, isolated lymphatic malformations, and benign lichenoid keratosis. The key distinguishing features are often found in clinical presentation, imaging, and molecular/genetic findings [4]. Treatment options range from conservative management to surgery depending upon the severity of the disease [5].

The present study aims to describe the characteristic imaging features of MDL, with emphasis on MRI findings, to aid in the diagnosis of this rare congenital overgrowth disorder. While several cases have been reported globally, only a few are from Asia. To our knowledge, this is the first case reported from Pakistan, notable for involvement of the lower limb with syndactyly and absence of nerve involvement along its course, distinguishing it from other Asian cases.

## 2 Case History/Examination

**FIGURE 1:**
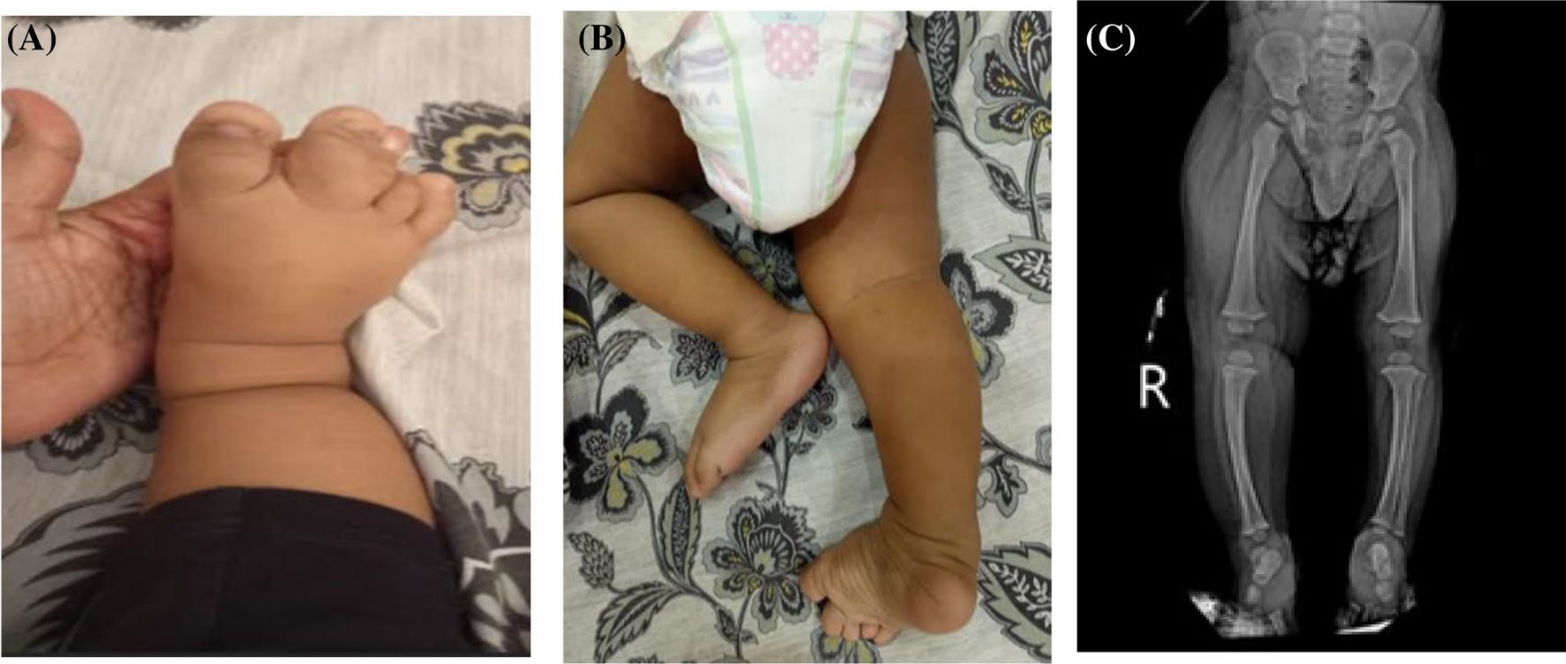
(A) Syndactyly of the right foot accompanied by localized gigantism of the right lower limb. (B) Prone‐position view demonstrating increased circumference of the right leg compared with the left. (C) Anteroposterior radiograph of the lower limbs showing increased soft‐tissue thickness on the right side, with no evidence of bony hypertrophy.

A 10‐month‐old boy presented to our institution with progressive, unilateral enlargement of the entire right lower extremity, which had been first noticed at 1 month of age. There was no history of trauma preceding the enlargement. The boy was born at term via cesarean section following an uncomplicated pregnancy. His birth weight and length were within normal limits and no abnormalities were noted at birth. Prenatal, postnatal and family histories were unremarkable. Clinical examination revealed hypertrophy of the right lower limb with associated syndactyly and a noticeable difference in circumference between the right and left lower limbs, as shown in Figures 1A and 1B. No significant bony growth was observed. The remainder of the clinical examination was unremarkable, and the child's weight and height were normal for his age, with appropriate developmental milestones. Imaging studies, including X‐rays, ultrasound and MRI, were performed. Based on the initial workup, a clinical diagnosis of MDL was suspected. The case was then discussed with a multidisciplinary team comprising a radiologist, plastic surgeon, and orthopedic surgeon.

## 3 Investigation and Treatment

X‐rays of the bilateral lower limbs, shown in Figure 1C, demonstrated no differences in the length or diameter of the femur, tibia, or fibula between the right and left sides. However, the lower right limb exhibited relatively increased soft tissue thickness compared to the left. Doppler ultrasound was performed to exclude vascular malformations, such as hemangiomatosis and lymphangiomatosis, and to differentiate the condition from vascular overgrowth syndromes like Klippel‐Trenaunay‐Weber syndrome. The ultrasound showed no evidence of vascular malformations and normal limb blood flow. To further assess soft tissue involvement, MRI was performed following the ultrasound.

**FIGURE 2:**
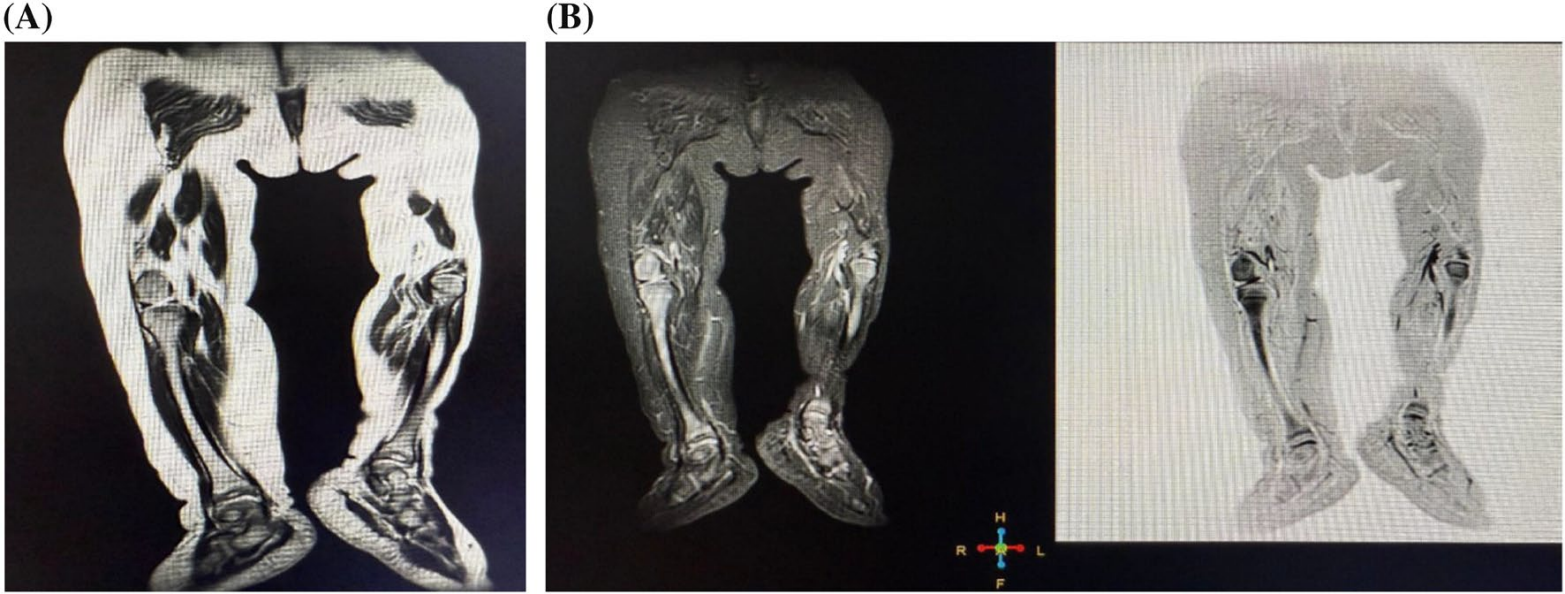
(A) Coronal T2‐weighted MRI of both lower legs demonstrating diffuse fat infiltration in the right lower leg. (B) Coronal STIR images showing appropriate fat suppression. No dilated vessels or varicosities are observed in the subcutaneous plane, and bone marrow signal intensity remains normal.

**FIGURE 3:**
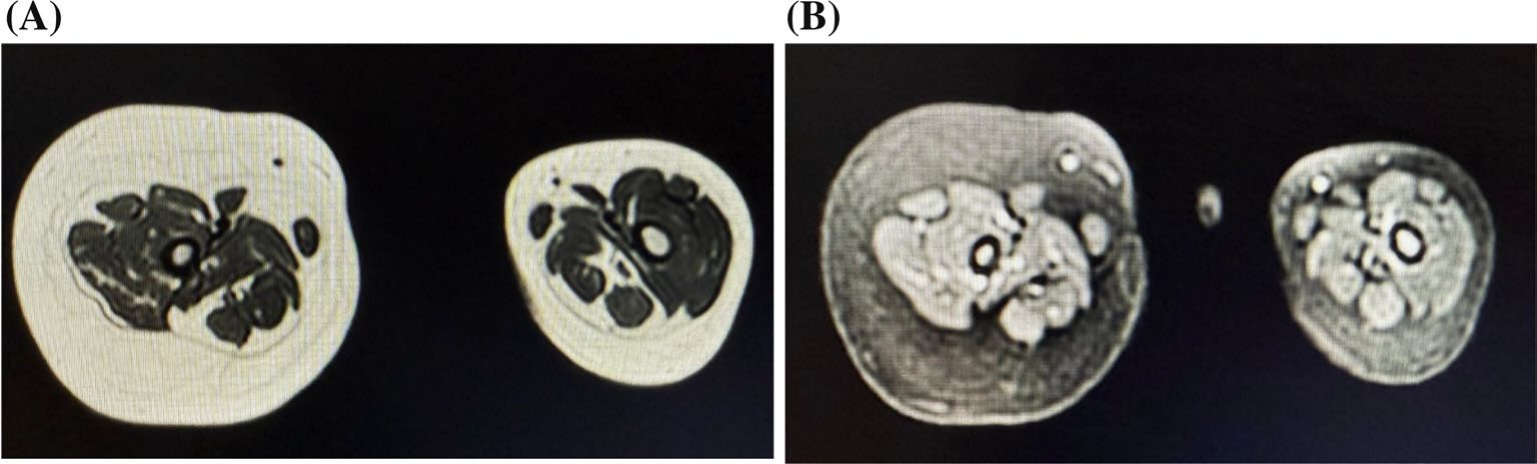
**(A)** Axial T2‐weighted MRI demonstrates normal muscle bulk and architecture with diffuse fat infiltration. **(B)** Axial STIR image showing adequate suppression of the fat signal.

MRI demonstrated non‐enhancing, diffuse fibrofatty hypertrophy in the subcutaneous plane of the right lower leg, with no nerve involvement and normal bone marrow, as shown in Figures 2A and 2B. The soft tissue hypertrophy appeared hyperintense on T1‐ and T2‐weighted images, with faint hypointense streaks on T1, involving the right thigh, lower leg, and foot. Axial images (Figures 3A and 3B) showed no evidence of vascular or lymphatic malformations. Based on clinical presentation and radiological findings, a diagnosis of MDL was established. The patient was counseled regarding treatment options, including surgical intervention and referred to a pediatric surgeon.

## 4 Discussion

We report a 10‐month‐old boy with unilateral right lower limb enlargement and syndactyly, diagnosed as MDL based on clinical and imaging findings. Interestingly, there was no nerve involvement along the course from the lumbosacral plexus, distinguishing this case from most reports in the literature, which describe MDL as an extreme form of nerve lipomatosis [6].

The pathogenesis of MDL is not fully understood, though recent studies suggest it falls within the spectrum of PIK3CA‐related overgrowth syndromes (PROS). Activating mutations in the PIK3CA gene result in abnormal mesodermal and ectodermal tissue proliferation, leading to localized hypertrophy, including fibrofatty overgrowth. MDL represents an isolated manifestation of PROS, in contrast to syndromic presentations such as CLOVES syndrome, Klippel‐Trenaunay syndrome, and fibroadipose overgrowth, which may involve vascular malformations or multiple organ systems [7].

Accurate diagnosis of MDL relies heavily on clinical and imaging features. Clinically, patients present with progressive limb enlargement, often noted at birth or in early infancy, and may have associated digital anomalies. In our patient, the right lower limb was enlarged with syndactyly, but limb function and neurovascular status were preserved. Imaging plays a central role in distinguishing MDL from other overgrowth syndromes. X‐rays typically show soft tissue hypertrophy without bony overgrowth, as in our case. Ultrasound is valuable for ruling out vascular malformations, such as hemangiomatosis or lymphangiomatosis, and differentiating MDL from vascular overgrowth syndromes like Klippel‐Trenaunay‐Weber syndrome characterized by disproportionate growth of soft and bony tissue of a body region (typically involving the lower extremities) and combined with cutaneous capillary, lymphatic, and venous malformations [8]. MRI remains the modality of choice, providing detailed characterization of fibrofatty tissue proliferation, extent of involvement, and relationship to surrounding structures. In our case, MRI demonstrated diffuse, non‐enhancing fibrofatty hypertrophy in the subcutaneous plane, with preservation of muscle architecture and absence of nerve involvement, findings consistent with MDL and crucial for diagnosis. Given below is the table to differentiate MDL from other causes of overgrowth syndrome [8]:

**Table:** 

Syndrome/Condition	Key Features	Distinct from MDL
Type I Macrodactyly	Isolated enlargement of digits (fingers/toes)	Limited to digits, unlike MDL which may involve entire limb segment
Muscle Fibrous/Adipose Hyperplasia (MH/FH)	Overgrowth of muscle/fibrous tissue, may have vascular anomalies (FAVA) or adipose overgrowth (FAO), can include HHML	May involve entire hemi‐body with multiple lipomas; MDL is usually localized to one limb
Facial Infiltrating Lipomatosis (FIL)	Adipose and soft tissue overgrowth localized to face, causing asymmetry	MDL typically affects limbs, not facial region
Epidermal Nevi (EN), Seborrheic Keratosis (SK), Benign Lichenoid Keratosis (BLK)	Cutaneous lesions	MDL is mesenchymal/fibrofatty overgrowth; skin lesions absent
CLOVES Syndrome	Congenital lipomatous overgrowth, vascular malformations, epidermal nevi, scoliosis/spinal anomalies; may have macrodactyly or wide extremities	MDL lacks systemic malformations and spinal involvement
Klippel–Trenaunay Syndrome (KTS)	Soft tissue and bony overgrowth with capillary, lymphatic, and venous malformations; varicosities or persistent embryonal veins	MDL has no vascular malformations
Megalencephaly‐Capillary Malformation (MCAP)	Macrocephaly, capillary malformations, body asymmetry, brain anomalies, neurodevelopmental delay	MDL confined to limb; no CNS involvement
Hemimegalencephaly (H‐MEG) / Dysplastic Megalencephaly (D‐MEG)	Cerebral overgrowth with cortical malformations	MDL is peripheral limb overgrowth, not cerebral

Based on growth patterns, a study of 38 cases classified MDL into three types: (a) nerve territory‐oriented, affecting the hand or foot along the median or medial plantar nerve distribution, with or without nerve involvement; (b) diffuse lipomatous, involving the entire extremity including all digits without nerve involvement; and (c) mixed pattern, showing nerve territory distribution in the hand or foot and diffuse involvement of the remaining limb [9]. Additionally, literature classifies MDL as static or progressive, depending on growth symmetry: static cases show proportional enlargement, while progressive cases exhibit disproportionate overgrowth [10]. Our case resembles the mixed pattern, with plantar nerve distribution involvement in the foot and diffuse enlargement of the lower limb.

Management of MDL depends on the severity of overgrowth and associated functional impairment. Conservative management may be appropriate for mild cases, whereas surgical intervention is indicated in patients with significant cosmetic or functional limitations. Procedures may include debulking of hypertrophic tissue or corrective surgery for syndactyly. Multidisciplinary evaluation, including radiology, pediatric surgery, and orthopedic input, is essential to guide optimal management and anticipate potential complications [11].

This case is particularly noteworthy as it represents the first reported instance of MDL from Pakistan and is distinguished by syndactyly and lack of nerve involvement, differentiating it from other cases in the Asian literature. Early recognition and appropriate imaging, especially MRI, are critical for accurate diagnosis, differentiation from other overgrowth syndromes, and timely referral for management.

## 5 Conclusion and Results

This is the first reported case of MDL in Pakistan. Reporting such rare cases enhances our understanding of the clinical presentation, progression, and treatment options for this condition, especially given the scarcity of prospective studies. MRI plays a crucial role in differentiating MDL from other overgrowth syndromes and neuropathies. Early diagnosis allows for better management planning, and documenting rare cases contributes to the advancement of therapeutic strategies and improved patient outcomes.

## Author Contributions

**Hafiza Sameeya Shehzadi:** conceptualization, data curation, investigation, writing – original draft. **Saumara Atif:** formal analysis, investigation, writing – review and editing. **Iqbal Hussain Dogar:** methodology, project administration, supervision, validation. **Usama Siddique:** resources, writing – review and editing. **Kamil Ahmad Kamil:** supervision, writing – review and editing.

## Funding

The authors have nothing to report.

## Ethics Statement

The authors have nothing to report.

## Consent

Written informed consent was obtained from the patient's parents to publish this case report and accompanying images in accordance with the journal's patient consent policy.

## Conflicts of Interest

The authors declare no conflicts of interest.

## Data Availability

Data sharing not applicable to this article as no datasets were generated or analyzed during the current study.
